# Raynaud's Disease—II

**Published:** 1908-12-05

**Authors:** 


### Medicine.

### RAYNAUD'S DISEASE—II.

Other lesions that may be associated with Raynaud's disease form quite a curious list, and the fact that they can all be associated directly with a disease whose pathology has been established as being a neurosis of the vaso-motor nerves seems to indicate that they also are objective manifestations of functional disorders.

Thus we have *hæmoglobinuria.* There are, of course, a great many cases of hæmoglobinuria that present no trace of Raynaud's disease, and there are a great many cases of Raynaud's disease in whom no sign of hæmoglobinuria is to be observed in any ordinary way; nevertheless, neither hæmoglobinuria nor Raynaud's disease is so common that the number of instances in which the two have been associated together can be attributed to mere chance. This applies equally well to the other conditions that may be associated with Raynaud's disease, and we need not repeat it in each instance.

The hæmoglobinuria is probably allied to the form known as paroxysmal hæmoglobinuria. It is not necessarily a permanent condition: indeed, quite the reverse; it may be present in girls at the time when the first symptoms of Raynaud's disease develop, and then disappear entirely though the Raynaud's disease advances. It is supposed by some that the hæmoglobinuria is due to the action of some intrinsic chemical substance or toxin, and it may be that a similar toxin lies at the root of Raynaud's disease.

*Purpura* is another condition that may be associated with Raynaud's disease. It is rarer than is hæmoglobinuria in this connection, but it is more striking. Probably the two are related to one another, but the purpura may be quite extensive without there being either hæmoglobinuria or hæmaturia, and *vice versa.* The occurrence of purpura, alarming though it is, by no means implies a bad prognosis. Recurrent attacks both of purpura and of Raynaud's disease may occur for years in young persons, and yet the purpura may presently cease to reappear in a way that reminds one of Henoch's purpura.

Another group of conditions that may be associated with Raynaud's disease are *cerebral* in type, though probably functional like the Raynaud's disease itself. They may take the form of transient vacancies of mind resembling petit mal, mental torpor, transient losses of consciousness, convulsive attacks of an hysterical nature, convulsive attacks of a true epileptiform type, passing hemiplegia, or aphasia, delirium, or apparent mania.

In other cases *sclerodermia* and *melanodermia* may occur. The sclerosis of the skin and subcutaneous tissues may begin in the face or on the trunk when the Raynaud's disease is present only in the hands, or the same parts may be affected both by the Raynaud's disease and by the sclerodermia. The fixation of the features and the deformity of the fingers and hands when the sclerodermic process extends across the joints, are precisely similar to those seen in cases of sclerodermia apart from Raynaud's disease.

Another condition seen in a fair number of cases of Raynaud's disease is *movable kidney* or even floating kidney (Byrom Bramwell). The connection between the two affections would strike one at first sight as being purely accidental; but in view of one line of treatment that has recently been tried with benefit the connection between the two would seem to be closer than one might have supposed. Lastly, it is noteworthy that some cases of *exophthalmic goitre* present symptoms not unlike mild Raynaud's disease

### Treatment.

The treatment of a case of Raynaud's disease should be essentially prophylactic; that is to say, the patient should be very careful indeed to avoid exposure to cold, undue fatigue, sudden excitement, and so forth. The hands should be washed in warm or hot water instead of cold; rooms should be well warmed, and, when possible, the patient should winter in a place where there are no frosts and no keen winds. Protection of the hands by warm gloves out of doors and by mittens indoors will often prevent an attack; indeed, there are a hundred and one little points which will become obvious when the case is before one, and when the pathology of the condition is understood.

When an attack is in progress treatment is indicated upon the same lines as that required in restoring the circulation in parts that have been exposed to extreme cold in healthy persons. Tissues that are in a state of local syncope or asphyxia should not be too suddenly exposed to great warmth; wrapping up in warm cotton wool, gentle active and passive movements, immersion in tepid water that is only slowly made warmer and warmer until it is hot, and so forth. It has been suggested that amyl-nitrite, nitro-glycerine, and other vaso-dilator drugs should bring relief, but this is not so in practice. The only drug which may be essential is morphia hypodermically to relieve the excruciating pain from which some patients suffer during recovery of the fingers or toes from the asphyxial stage of an attack; yet this should be withheld whenever possible, because the disease is not a fatal one, and it would be dreadful to have the morphia habit developed in addition to the disease.

Supposing the third stage of the complaint to have been reached—local gangrene—treatment will be by hot boric acid fomentations and the like in the ordinary surgical way. Sloughs may need to be removed by means of scissors, but as a rule the gangrene affects only small areas of tissue at a time, and fomentations will effect a cure. Calcium chloride does so much good in cases of bad chilblains that it would probably be of benefit in these cases also. Doses of 10 grains three times a day in capsules would be suitable. Amputation of parts of fingers is fortunately seldom needed unless as the result of inefficient care on the patient's part when the sore places first develop.

Four other points in treatment deserve special mention. First, there is no doubt that not a few cases improve when ·naphthol in 10-grain doses or sodium sulphocarbolate in 20-grain doses are given orally; these seem to be of particular benefit in cases complicated by sclerodermia—subjective benefit anyway, even if not objective.

Secondly, many of the patients attribute relief to electrical treatment of various kinds, of which the two best known are (*a*) immersion of the hands or other affected parts in a salt-water bath through which a constant current that can be interrupted periodically is being passed; and (*b*) faradism over the spine at the level of the origin of the vaso-motor nerves supplying the affected parts. This electrical treatment is chiefly employed between attacks and not during them, the number of sittings and the duration of each varying in different cases.

Thirdly, Dr. Cushing, an American surgeon, some little time ago recommended another method of treatment, namely, bandaging the affected limb with an elastic bandage or tourniquet with the object of shutting off the arterial circulation for some minutes from the affected parts. On removing the constriction the blood flows freely owing to the vaso-motor relaxation. In cases in which the spasm is severe, the application of the tourniquet or bandage may be repeated several times at frequent intervals before the vaso-constriction in the parts is overcome, and the normal temperature and colour return to them. Dr. Cushing reports marked benefit in several cases of Raynaud's disease from this plan of treatment.

Lastly, Dr. George Gibson has reported two cases in which extreme mobility of a kidney was associated with Raynaud's disease, and in which very marked benefit as regards the latter resulted from stitching the kidney to the abdominal wall. Dr. Byrom Bramwell, though at first very sceptical as to whether the improvement was the direct result of the treatment or not, confirms the fact that stitching a movable kidney may result in great amelioration of the symptoms of Raynaud's disease. The mode of action here is by no means obvious, but if the fact is confirmed in any large proportion of cases it is clear that nephropexy may be a thing to advise rather than otherwise in cases of Raynaud's disease.

